# Association between orthopedic manifestations and tethered cord release in patients with spina bifida: a survival analysis

**DOI:** 10.1007/s00381-025-06837-x

**Published:** 2025-05-17

**Authors:** Anthony M. Padgett, Abigail Nishikawa, Jun Kit He, Jeffrey P. Blount, Anastasia Arynchyna-Smith, Betsy Hopson, Gerald McGwin, Brandon G. Rocque, Michael J. Conklin

**Affiliations:** 1 Division of Pediatric Orthopaedic Surgery, University of Alabama at Birmingham, Birmingham, AL USA; 2 University of Alabama at Birmingham Heersink School of Medicine, Birmingham, AL USA; 3 Division of Pediatric Neurosurgery, University of Alabama at Birmingham, Birmingham, AL USA; 4 Department of Epidemiology, University of Alabama at Birmingham, Birmingham, AL USA

**Keywords:** Spina bifida, Tethered cord, Orthopedic, Survival

## Abstract

**Purpose:**

Patients with spina bifida (SB) are at risk for symptomatic tethered cord syndrome (TCS). Orthopedic decline, a common manifestation of TCS, is an indication for tethered cord release (TCR). Our objective is to determine if patients with SB who have undergone specific orthopedic operations (release of hip or knee contracture and correction of cavus foot) require TCR at a higher rate than those not undergoing these operations.

**Methods:**

An institutional database was queried to identify all children with SB from 2009 to 2022. Data included functional level of lesion (FLOL), ambulatory status, and diagnosis of myelomeningocele (MMC) vs. closed neural tube defects. Survival analysis was performed to test the association between TCR and index orthopedic operations. Kaplan–Meier survival curves and multivariate Cox proportional hazard models were generated.

**Results:**

There were 659 patients. Thirty-four (5.2%) had a history of orthopedic operation, and 625 (94.8%) had no history of orthopedic operation either before TCR or at last follow-up. Three of thirty-four (8.6%) in the orthopedic group underwent TCR after the orthopedic operation. Two hundred two of six hundred twenty-five (32.3%) in the non-orthopedic group underwent TCR. The adjusted hazard ratio (controlling for FLOL, ambulation, and diagnosis) was 3.8 (95% confidence interval 1.2–11). In MMC, the hazard ratio for the non-orthopedic group compared to the orthopedic group was 5.05 (95% confidence interval 1.2–20.7) which was significant.

**Conclusion:**

Patients with MMC who underwent the specific orthopedic operations were significantly less likely to have subsequent TCR surgery. One possible explanation is that lower extremity deformity correction may alter surgeon behavior regarding TCR.

**Supplementary Information:**

The online version contains supplementary material available at 10.1007/s00381-025-06837-x.

## Introduction

Neural tube defects refer to congenital abnormalities of the brain or spinal cord that result from failure of normal neurulation. They are the most common group of congenital central nervous system malformations with a worldwide incidence of approximately 1.0 to 10.0 per 1000 births [1]. In patients with SB, there are abnormalities in the embryologic processes that give rise to the caudal spinal cord, meninges, and overlying tissues, the most common and severe of which is myelomeningocele (MMC) [2].

Symptomatic TCS is thought to be caused by abnormal stretching of the cord due to adhesions of congenital or acquired origin attaching the spinal cord to the overlying tissues. This mechanical insult leads to spinal cord ischemia, diminished glucose utilization, a shift from oxidative to anaerobic metabolism, and metabolic failure [3, 4]. The lifetime incidence of TCS is approximately 0.25 per cases 1000 births, with the largest percentage attributed to patients with SB [5]. Surgical management of TCS, TCR, is performed in 10–32% of MMC patients over the course of a lifetime [3].

Clinical features of TCS include but are not limited to lower back pain, sensory and motor deficits, incontinence, and orthopedic manifestations such as spasticity of the lower extremities, contractures, and pes cavus [6]. In patients without SB, the diagnosis of TCS is highly dependent on the combination of clinical symptoms, physical exam findings and magnetic resonance imaging [7, 8]. However, for patients with SB who have had surgical repair of their neurological defect, imaging is unreliable as the spinal cord appears tethered in nearly all cases, whether or not there are clinical symptoms [9]. Additionally, the manifestations of TCS can be insidious and parallel deficits associated with SB, making new or progressive symptoms difficult to identify [10]. Due to the increased risk of developing TCS in individuals with SB and the ambiguity associated with the diagnosis, further elucidation of the clinical manifestations of TCS is needed.

In patients with a known history of SB, the most common manifestations of orthopedic decline indicating potential TCS are decreased lower extremity strength, functional change in ambulation, back pain, leg pain, and increased leg or foot deformity [11, 12]. While these orthopedic signs and indications for TCR have been established, the temporal association between the two has not been well examined [7, 13]. Previous literature has shown that patients with SB who undergo TCR frequently require subsequent orthopedic surgery [13]. The current study aims to determine if patients with SB who undergo specific orthopedic procedures: correction of a cavus foot deformity or release of hip or knee contractures, are more likely to subsequently undergo TCR. Since in some unknown percentage of patients these orthopedic deformities may represent manifestations of TCS, and orthopedic surgery only treats the peripheral manifestations of TCS, we hypothesized that individuals who undergo these orthopedic corrections are at increased risk for subsequent TCR surgery.

## Methods

The Institutional Review Board approved the collection of all data used in this study. A comprehensive institutional database assembled using National Spina Bifida Patient Registry (NSBPR) methodology was queried to retrospectively identify all children with SB between 2009 and 2022. All patients with the primary diagnosis of SB could be enrolled in the NSBPR; details of the enrollment process have been previously described [12]. Our institution has been included in the NSBPR since its inception, and we have maintained 99% enrollment of all eligible participants.

**Fig. 1:**
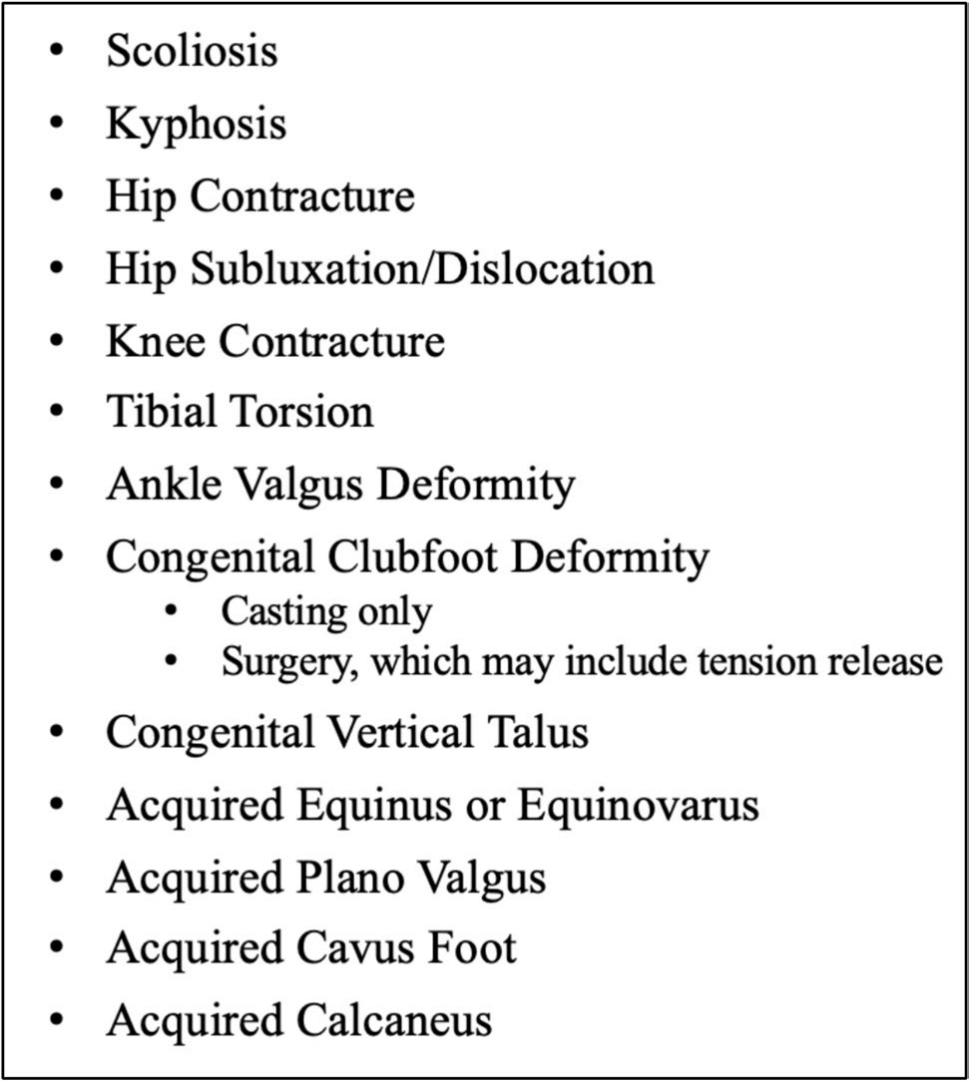
List of orthopedic surgical procedures recorded in the NSBPR

Data gathered included demographics, diagnosis (MMC versus other), and clinical outcomes. Diagnoses other than MMC include meningocele, terminal myelocystocele, lipomyelomeningocele, fatty/thickened filum/low lying cord, and split cord malformation. Clinical data included functional level of lesion and ambulatory status [14]. Patients who underwent a cavus foot correction (Fig. 1) or operation for hip or knee contracture were categorized as the orthopedic group. Patients who did not undergo one of these procedures for purposes of this study are delineated as the non-orthopedic group. As many individuals with SB have musculoskeletal deformities such as clubfeet, congenital vertical talus, and tibial torsion that most likely are not related to TCS, these procedures were not chosen. Furthermore, since scoliosis is only captured in the database if patients undergo surgical correction, this deformity was not chosen as an orthopedic indicator. The complete list of surgically treated orthopedic conditions captured by the NSBPR is shown in Fig. 1.

Indications for orthopedic surgical intervention for cavus foot and hip or knee contracture are standard at our institution. In ambulators, contractures or deformities that are significant enough to interfere with or preclude bracing and leading to alterations or inefficiencies in gait are considered for operative correction after neurosurgery has confirmed that a TCR is not necessary. In non-ambulators, surgery is considered for deformities leading to skin breakdown or impending skin breakdown or causing difficulties with positioning or care.

Neurosurgical indications for TCR are standard at our institution and include intractable back or leg pain, decline in bladder function documented on urodynamic testing, and documented increase in lower extremity weakness. However, increasing lower extremity deformity that might be related to TCS is not considered a strict surgical indication in the absence of the above indications.

It should also be noted that for the purposes of the current study, and per the instructions for data acquisition in the NSBPR manual, the initial neurosurgical operation is considered a repair of the lesion, not a TCR. This holds true for all categories of SB captured by the NSBPR. Therefore, TCR in this study implies a second surgical procedure.

### Statistical analysis

Primary outcome was defined as the time from first clinic visit to surgical TCR. Patients who did not undergo a TCR during the study period were treated as censored, with the time to the most recent visit date as the censoring time. Categorical variables were compared using the chi-squared test. Kaplan–Meier curves with a primary endpoint of time to first TCR were constructed to compare survival time between the orthopedic group and the non-orthopedic group. Adjusted multivariate Cox proportional hazards models with 95% confidence intervals (CI) were generated to evaluate for independent association between the orthopedic intervention and TCR. A hazard ratio above 1.0 represents increased risk of TCR. The influence of ambulation status, functional level, diagnosis, and demographic characteristics were also examined. For non-MMC diagnoses, because of the small number of individuals in each category, these were grouped together and compared to MMC. All hypothesis tests were two-sided; a *p* value of 0.05 was considered significant for analyses. We then performed separate multivariate Cox proportional hazards models for each diagnosis, MMC and other (closed neural tube defects).

## Results

### Demographics

Six hundred fifty-nine patients met inclusion criteria for the study. Four hundred eighty-eight patients had MMC while 171 patients had closed neural tube defects. Thirty-four (5.2%) of patients had undergone one of the index orthopedic procedures and 625 (94.8%) had not. Significant differences between the two groups were present for functional level of lesion (*p* < 0.04) and diagnosis (MMC more common in the orthopedic group, *p* < 0.01). Other demographic variables were similar between the two groups (Table 1).

**Table 1:** Characteristics of patients with a history of orthopedic intervention compared to those with no prior orthopedic operation

Characteristic	Orthopedic group (*n* = 34) (%)	Non-orthopedic group (*n* = 625) (%)	*P*-value
**Average age** (months)	152.3 ± 60.9	120.2 ± 95.5	> 0.05
**Functional level of lesion**			
Thoracic High lumbar Mid lumbar Low lumbar Sacral	5 (14.7%) 5 (14.7%) 12 (35.3%) 7 (20.6%) 5 (14.7%)	137 (21.9%) 42 (6.7%) 163 (26.1%) 73 (11.7%) 210 (33.6%)	** < 0.04**
**Ambulatory status**			
Community ambulator Other ambulatory status	16 (47.1%) 18 (52.9%)	311 (49.8%) 314 (50.2%)	0.76
**Sex**			
Male Female	15 (44.1%) 19 (55.9%)	296 (47.4%) 329 (52.6%)	0.71
**Race**			
White Non-white	25 (73.5%) 9 (26.5%)	448 (71.7%) 177 (28.3%)	0.82
**Diagnosis**			
Myelomeningocele	32 (94.1%)	456 (73.0%)	
Other	2 (5.9%)	169 (27.0%)	**< 0.01**
▪ Lipomyelomeningocele	▪ 2	▪ 135	
▪ Split cord malformation	▪ 0	▪ 34	

### Tethered cord release

**Fig. 2:**
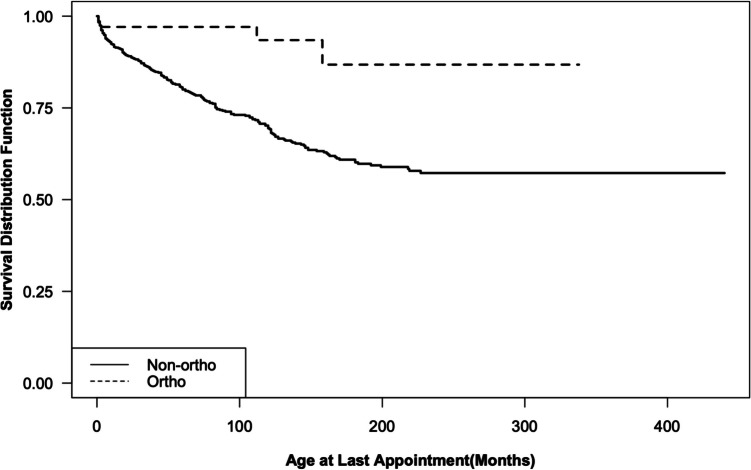
Kaplan–Meier survival curves with time to first tethered cord release among patients with a history of orthopedic intervention (dashed) compared to those with no prior orthopedic operation (solid)

Three (8.8%) of the 34 patients in the orthopedic group subsequently underwent TCR (no patients in the orthopedic group had undergone TCR prior to the index orthopedic procedure). 149 (23.8%) of the 625 patients in the non-orthopedic group had a history of TCR (Table 1). The Kaplan–Meier survival curves for the two groups are shown in Fig. 2. Kaplan–Meier survival curves are shown separately for MMC (Fig. S3) and other diagnoses (Fig. S4). in the supplemental data.

Data for the adjusted multivariate Cox proportional hazards models is shown in Table 2. The hazard ratio for the non-orthopedic group compared to the orthopedic group was 3.8 (95% confidence interval 1.2 to 12.0, *p* = 0.02), demonstrating that the association between the index orthopedic interventions and TCR is independent of the other variables. Diagnosis other than MMC was an independent risk factor for TCR with a hazard ratio of 4.2 (95% confidence interval 3.1 to 5.7, *p* < 0.001). Significant associations were not observed between TCR and other variables.

**Table 2:** Cox proportional hazard regression analysis for time to first TCR

Variable	Hazard ratio (95% CI)	*P*-value
No history of index orthopedic operation	**3.8 (1.2, 12.4)**	**0.02**
Race	1.2 (0.9, 1.6)	0.3
Sex	0.9 (0.7, 1.2)	0.5
Diagnosis (other than MMC)	**4.2 (3.1, 5.7)**	** < 0.0001**
**Functional level of lesion**		
Thoracic High lumbar Mid lumbar Low lumbar Sacral	1.3 (0.8, 2.2) 1.8 (0.9, 3.6) 1.4 (1.0, 2.1) 1.2 (0.6, 1.7) 1.1 (0.8, 2.1)	0.3 0.08 0.09 0.9 0.4
Ambulation status	1.4 (1.0, 2.2)	0.07

*CI*, confidence interval; *MMC*, myelomeningocele

When separately evaluating patients with MMC using multivariate Cox proportional hazards models, the hazard ratio for the non-orthopedic group compared to the orthopedic group was 5.05 (95% confidence interval 1.2–20.7, *p* = 0.02) which was significant. In patients with other neural tube defects, the hazard ratio for the non-orthopedic group compared to the orthopedic group was 1.98 (95% confidence interval 0.26–14.85, *p* = 0.51) demonstrating that there was nearly a twofold increased hazard, though it was not statistically significant.

## Discussion

In individuals without SB, TCR is indicated in children who exhibit motor and sensory dysfunction, deterioration of gait, bladder dysfunction, and back or leg pain and foot deformities [6]. Early intervention is recommended to avoid irreversible damage and lifelong neurological complications [15]. In the setting of SB, the diagnosis of TCS requires clear clinical deterioration from baseline neurological level, often monitored in a multidisciplinary clinic [16]. Typical orthopedic manifestations include lower extremity deformities such as pes cavus, lower extremity contractures, back pain, leg pain, decline in ability to ambulate, and scoliosis [17]. In children with SB, who have already existent neurologic deficits, gait disturbances and lower extremity deformities, it can be difficult to tell whether alterations are due to the natural history of the neurologic deficits or due to TCS. In a recent survey of pediatric neurosurgeons using expert consensus methodology, the strongest clinical indicators for TCR in patients with known SB were deterioration in urinary continence, followed by decreased leg strength or sensation, functional change in ambulation, deterioration in bowel continence, and increased leg or foot deformity [11]. However, only 3.8% of respondents indicated that increasing leg or foot deformity was an absolute indication for TCR, as opposed to 21.3% for urinary continence deterioration and 19% for decreasing leg strength/sensation. Therefore, it would be useful to have a more thorough understanding of the relationship between specific musculoskeletal deformities and TCS.

The current study was undertaken to determine if patients followed in a multidisciplinary SB clinic who underwent operations for specific orthopedic deformities (cavus feet, knee, or hip contractures) would subsequently be diagnosed with TCS and undergo surgical TCR at a higher rate than those who did not undergo these orthopedic corrections. In our multidisciplinary clinic, direct communication between orthopedics and neurosurgery is standard. If the orthopedist detects neurologic decline or these specific deformities, then neurosurgery is made aware of these issues. In the absence of neurologic decline, the conclusion is that there are not enough clinical findings to move forward with TCR and the patient undergoes orthopedic surgical correction. We were curious as to whether these patients then subsequently underwent TCR, and our original hypothesis was that they would undergo TCR at a higher rate. Therefore, the results of this study were unexpected. Individuals who underwent the index orthopedic procedures underwent subsequent TCR less commonly than the non-orthopedic group. This was statistically significant even when controlling for other variables using the multivariate Cox proportional hazards model. Interestingly, when separately evaluating individuals with MMC compared to other closed neural tube defects, this effect was statistically more powerful in the MMC group. For the group with closed neural tube defects there was a nearly twofold increased hazard. Therefore, though it was not statistically significant, there was a positive association.

These results demonstrate an inverse association between specific orthopedic operations and subsequent TCR in the SB population. These findings in no way implicate that the orthopedic intervention offers protection from TCS. Rather, the musculoskeletal findings may be sufficient to impact neurosurgical decision-making. The diagnosis of TCS is inherently clinical, multi-factorial, and subjective. Imaging studies characteristically show fixed, low position conus, and presence of fat in lipomatous anomalies. Functional studies such as urodynamics and gait analysis may contribute but are known to demonstrate significant variance and lack of objectivity [18, 19]. As such, the presence of musculoskeletal findings such as scoliosis, contractures, and cavus foot may promote a clinical diagnosis of TCS particularly if they appear progressive. This data analysis suggests that treatment of these findings by orthopedic intervention does result in fewer TCS diagnoses and TCRs. Given that the collective morbidity and risks from orthopedic interventions may be less than TCR there may be a role for treating the musculoskeletal problems and observing closely for progressive decline that is the clinical hallmark of TCR. If progression is not observed, then the patients can be potentially spared the morbidity of TCR provided they remain under close, continued observation [3, 12, 20]. The results of the current study may prove useful for both orthopedic surgeons and neurosurgeons managing this challenging condition.

For purposes of the current study, not all orthopedic operations were considered “index orthopedic procedures.” Foot deformities such as clubfeet, congenital vertical talus, and calcaneus foot deformity are common in SB and not thought to be indicative of TCS [21]. These deformities are either congenital (clubfoot and congenital vertical talus) or expected developmental deformities due to muscle imbalance (calcaneus foot deformity). However, spasticity leading to progressive joint contracture or progressive cavus or cavovarus foot deformities may be indicative of TCS. Though the authors would have liked to examine the relationship between scoliosis and TCS, this was not feasible using the NSBPR as it only tracks orthopedic deformities that undergo an operation (Fig. 1). With current trends in non-operative treatment of scoliosis in SB [22, 23] and the high prevalence of scoliosis in this population [24], it was not possible to explore this relationship.

It was also noteworthy that children with lesions other than MMC were more likely to undergo TCR. This is likely because lipomyelomeningocele, which has a high probability of retether, is included in the non-MMC group [25, 26].

### Limitations

The current study has several limitations. The NSBPR is a registry of patients who consent to enrollment and are followed in the multidisciplinary SB clinic. The NSBPR enrollment rate at our institution is 99%, therefore inclusion bias at our institution should not be a factor. Nevertheless, the results may have limited external validity. Second, the NSBPR only collects data on orthopedic deformities that underwent an operation. Therefore, cavus feet or joint contractures that did not rise to the level of needing surgery are not tracked. Third, the inclusion of operations for cavus foot and hip or knee contracture and exclusion of other orthopedic procedures may appear arbitrary. However, individuals with SB manifest an abundance of orthopedic deformities. For purpose of the current study, the authors chose those that were not congenital but are likely to develop over time due to spasticity or increasing tone. Lastly, the indications for TCR are not standardized nor decided a priori.

## Conclusion

In a large, single-institutional study using NSBPR data, the relationship between three specific orthopedic procedures and subsequent TCR in patients with SB was investigated using survival analysis. It was noted that individuals who underwent the index orthopedic procedure(s) were less likely to undergo subsequent TCR than those who did not undergo these orthopedic procedures. This was statistically significant even when controlling for other variables using the multivariate Cox proportional hazards model. Further research could include a larger sample size involving all the clinics participating in the NSBPR to determine if this data is generalizable on a larger scale.

## Supplementary Information

Below is the link to the electronic supplementary material.

Supplementary file1 (DOCX 182 KB )

## Data Availability

No datasets were generated or analysed during the current study.
