# Supplementary material for: Association between orthopedic manifestations and tethered cord release in patients with spina bifida: a survival analysis
Source: Childs Nerv Syst. 2025 May 17;41(1):186. doi: 10.1007/s00381-025-06837-x (PMC12085327; doi:10.1007/s00381-025-06837-x)

**Figure 3**. Kaplan Meier survival curves with time to first tethered cord release among patients with a history of orthopaedic intervention (dashed) compared to those with no prior orthopaedic operation (solid) for MMC diagnosis


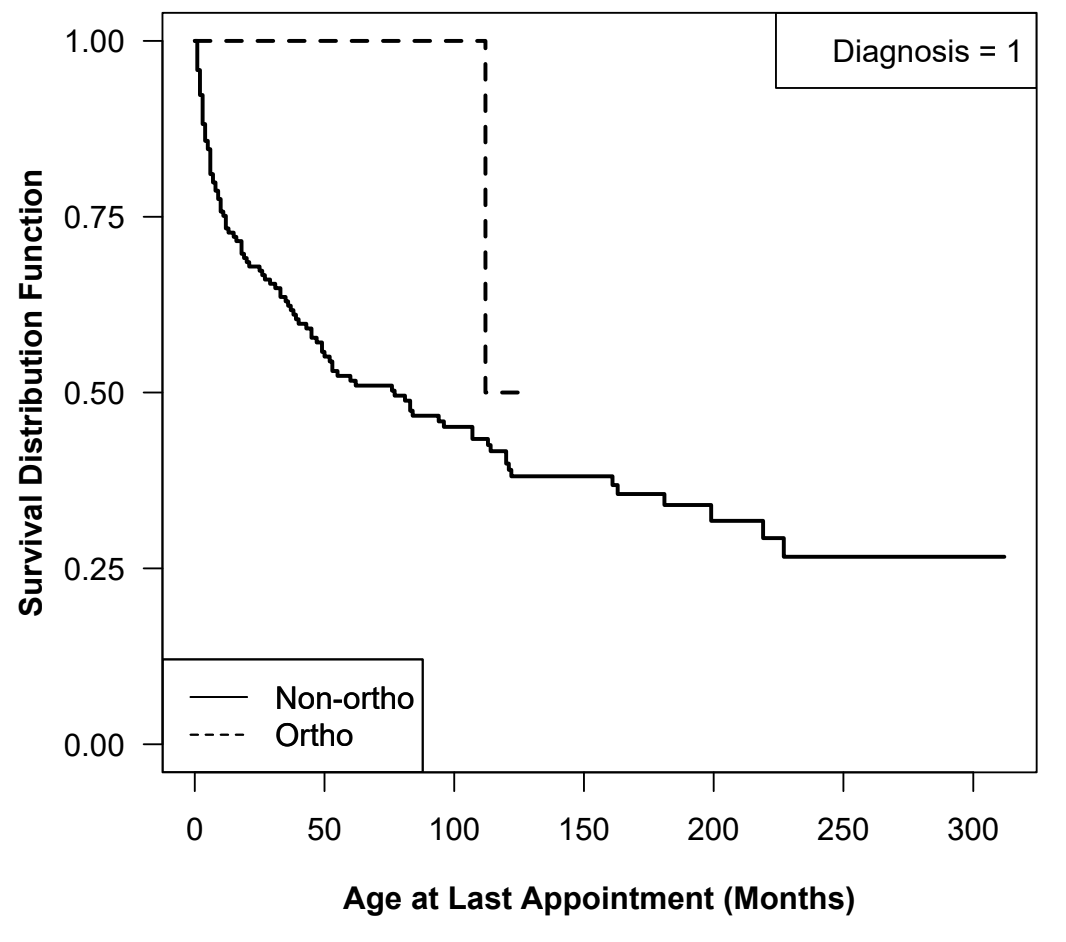


**Figure 4**. Kaplan Meier survival curves with time to first tethered cord release among patients with a history of orthopaedic intervention (dashed) compared to those with no prior orthopaedic operation (solid) for “other” diagnosis


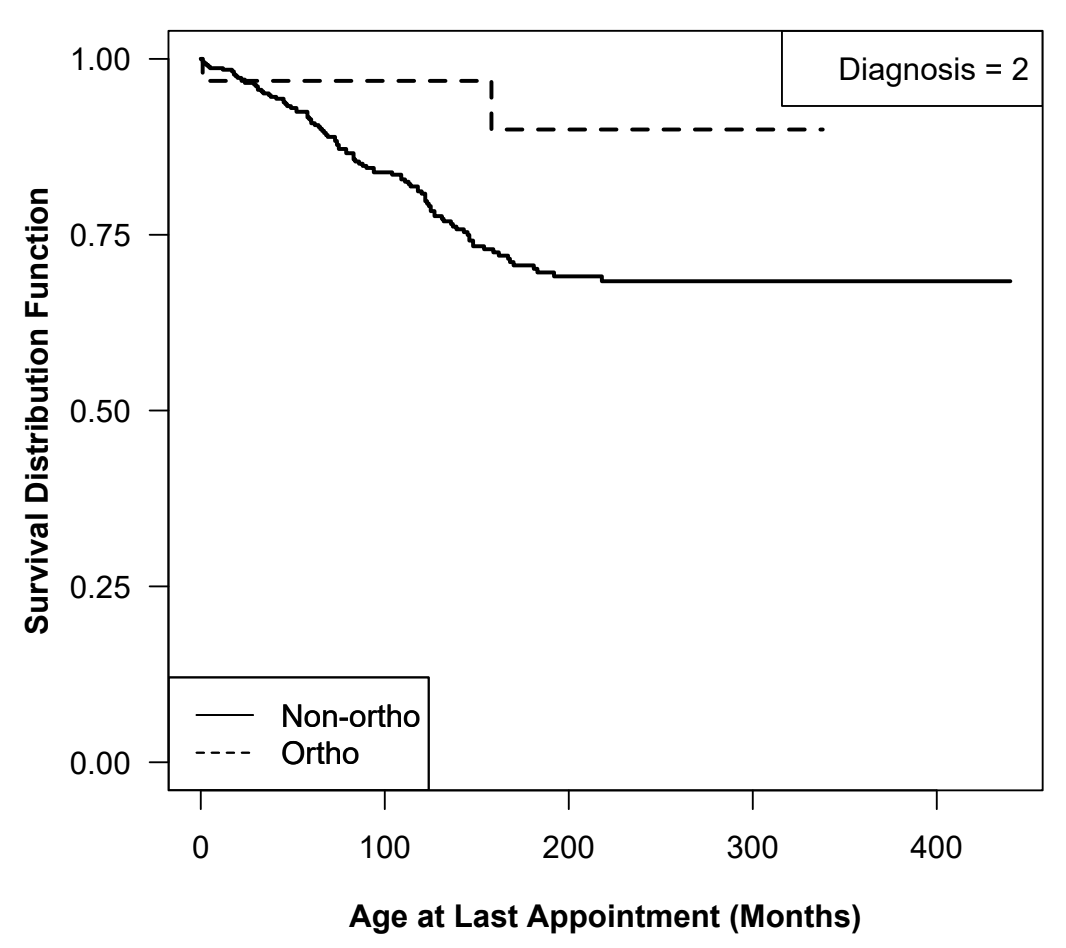

Supplement: Supplementary file 1 — Supplementary file1 (DOCX 182 KB ) [file 381_2025_6837_MOESM1_ESM.docx]
